# Supplementary material for: Generation of Orthotopic Patient-Derived Xenografts in Humanized Mice for Evaluation of Emerging Targeted Therapies and Immunotherapy Combinations for Melanoma
Source: Cancers (Basel). 2023 Jul 20;15(14):3695. doi: 10.3390/cancers15143695 (PMC10377652; doi:10.3390/cancers15143695)
Supplement: Supplementary file 1 [file cancers-15-03695-s001.zip › cancers-2455654-supplementary.pdf]

Supplementary Material

Generation of Orthotopic Patient-Derived Xenograft in Humanized Mice for Evaluation of Emerging Targeted Therapies and Immunotherapy Combinations for Melanoma

Chi Yan, Caroline A. Nebhan, Nabil Saleh, Rebecca Shattuck-Brandt, Sheau-Chiann Chen,

Gregory D. Ayers, Vivian Weiss, Ann Richmond*, Anna Vilgelm*

*** Correspondence:**

Ann Richmond, PhD, Email: ann.richmond@vanderbilt.edu

Anna Vilgelm, MD, PhD, Email: Anna.Vilgelm@osumc.edu

Supplementary figures

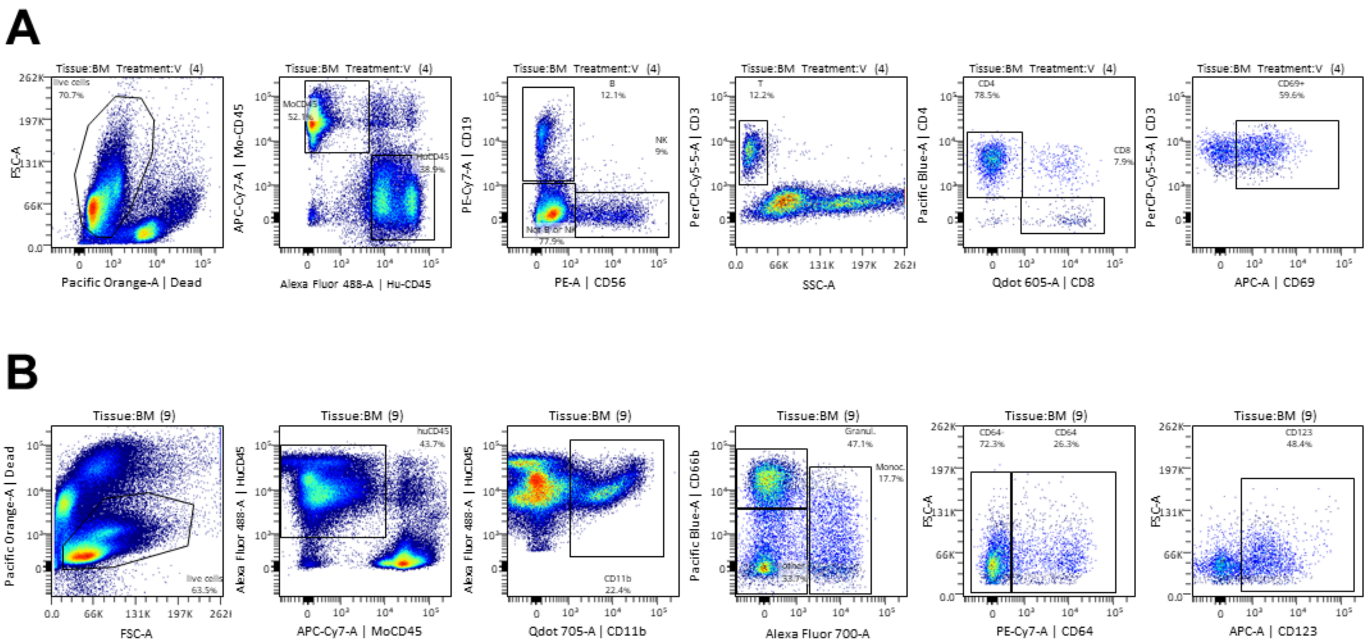

Supplementary Figure S1. Gating strategy to identify key immune subsets in huNSGS mice. A. Representative cytometry plots showing lymphoid immune subsets gating in huNSGS mice. **B.** Representative cytometry plots showing myeloid immune subsets gating in huNSGS mice.

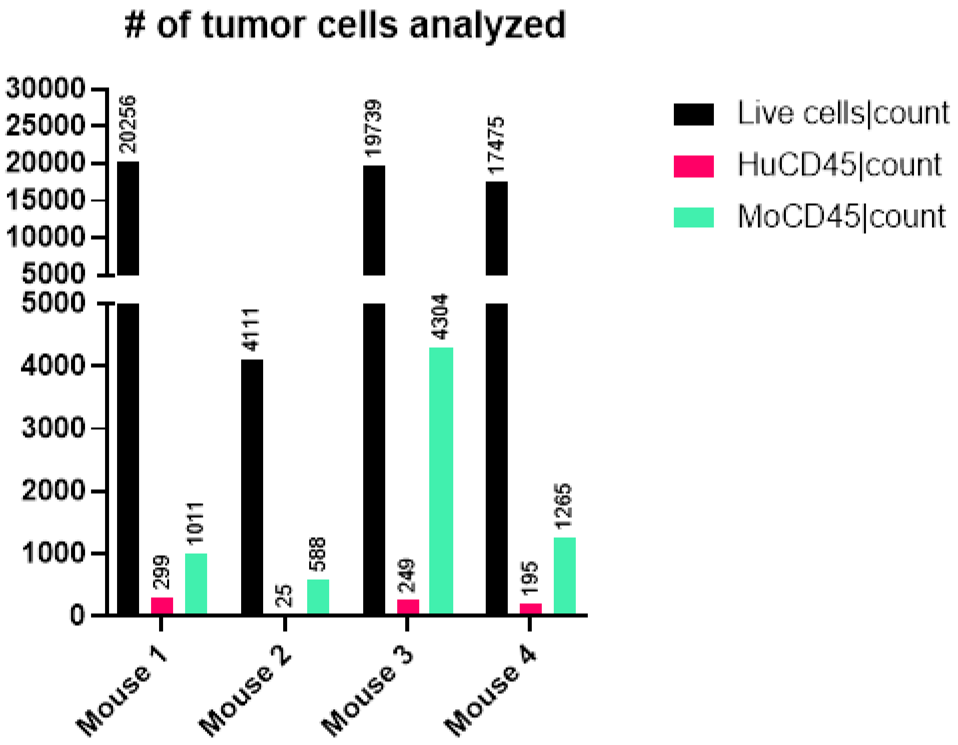

Supplementary Figure S2. Absolute numbers of live cells, human CD45 cells, and mouse CD45 cells from the flow cytometry analysis of tumors grown in huNSGS mice shown in Figures 2F and G.

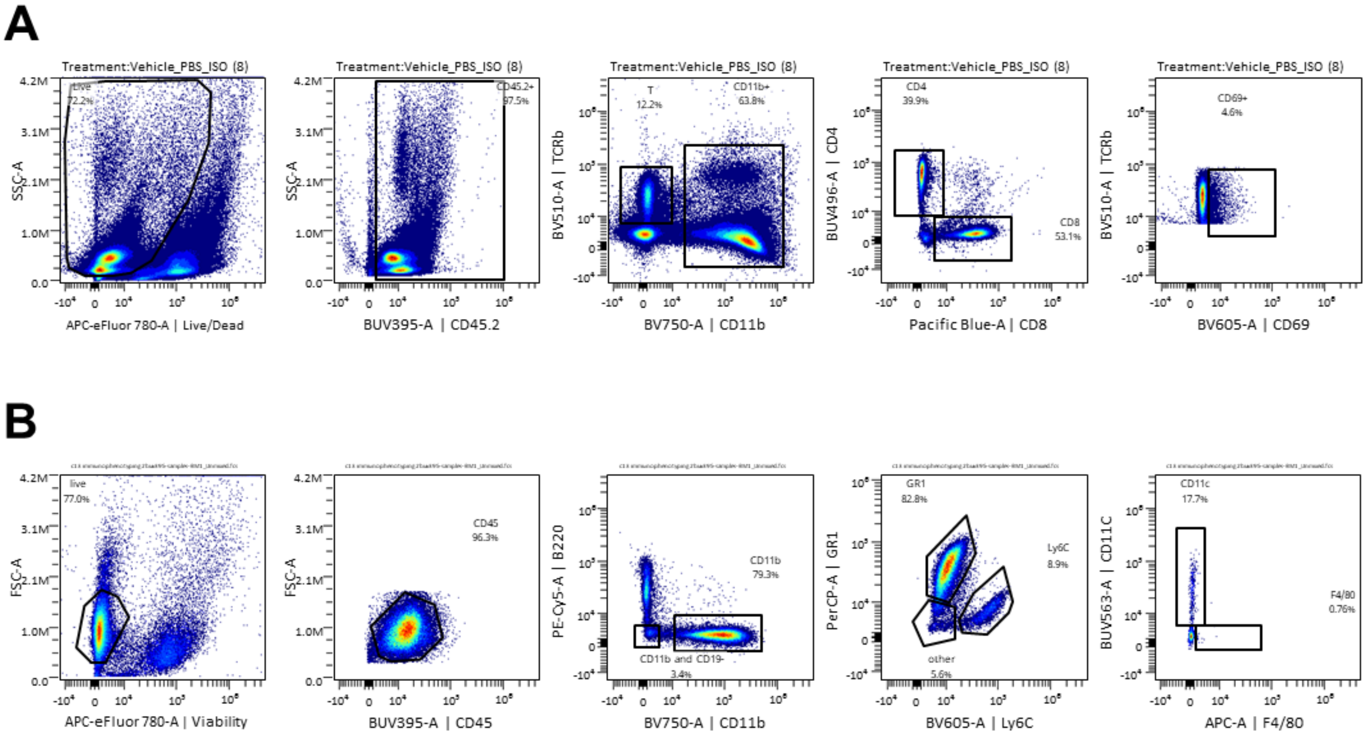

Supplementary Figure S3. Gating strategy to identify immune cell subsets in immunocompetent mice. A. Representative cytometry plots showing lymphoid immune subsets gating in wild type C57Bl/6 mice. **B.** Representative cytometry plots showing immune subsets gating in wild type Balb/C mice.

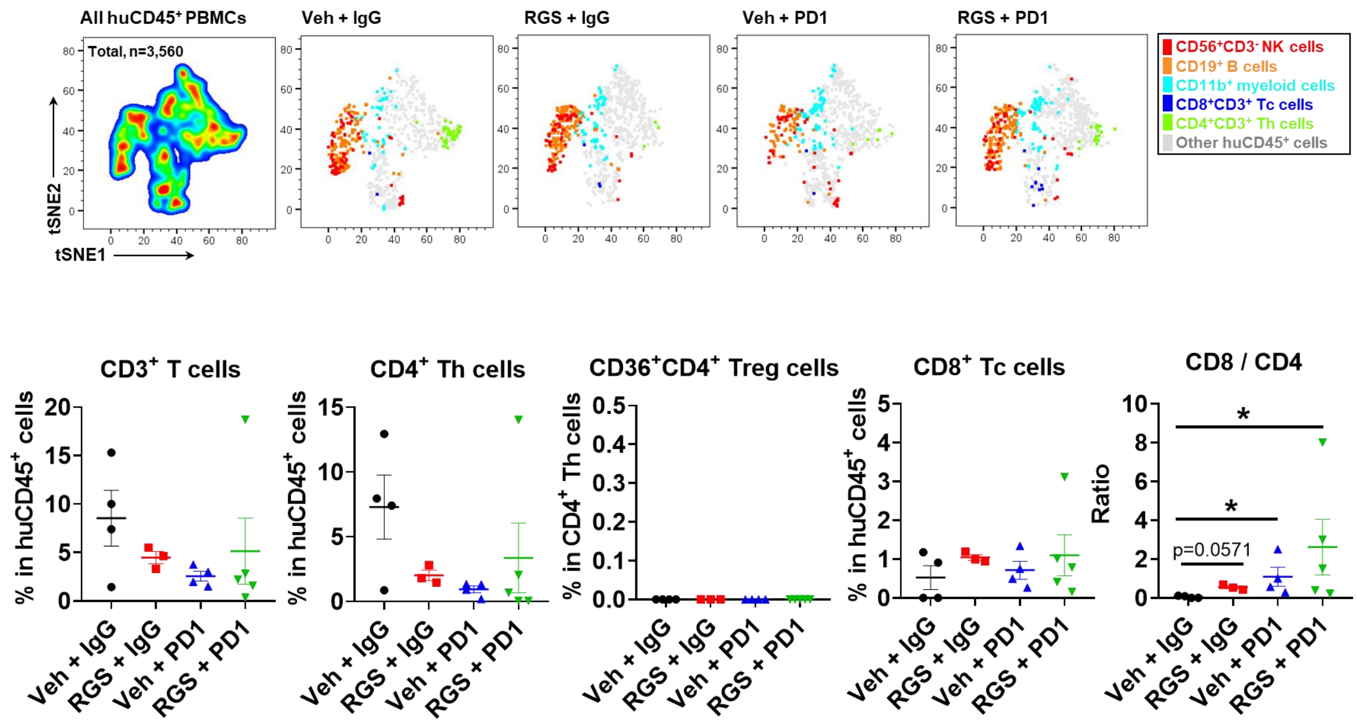

Supplementary Figure S4. Rigosertib plus anti-PD-1 induced CD8/CD4 cell ratio in the blood of huNOG-EXL-PDX mice bearing PDX-3101 tumors. tSNE analysis of immune profile and percentages of CD3+ T cells, CD4+CD3+ helper T (Th) cells, CD36+CD4+CD3+ regulatory T (Treg) cells, CD8+CD3+ cytotoxic T (Tc) cells, and CD8/CD4 ratio in the blood of individual humanized mice. Cells were gated on human CD45+ cells.

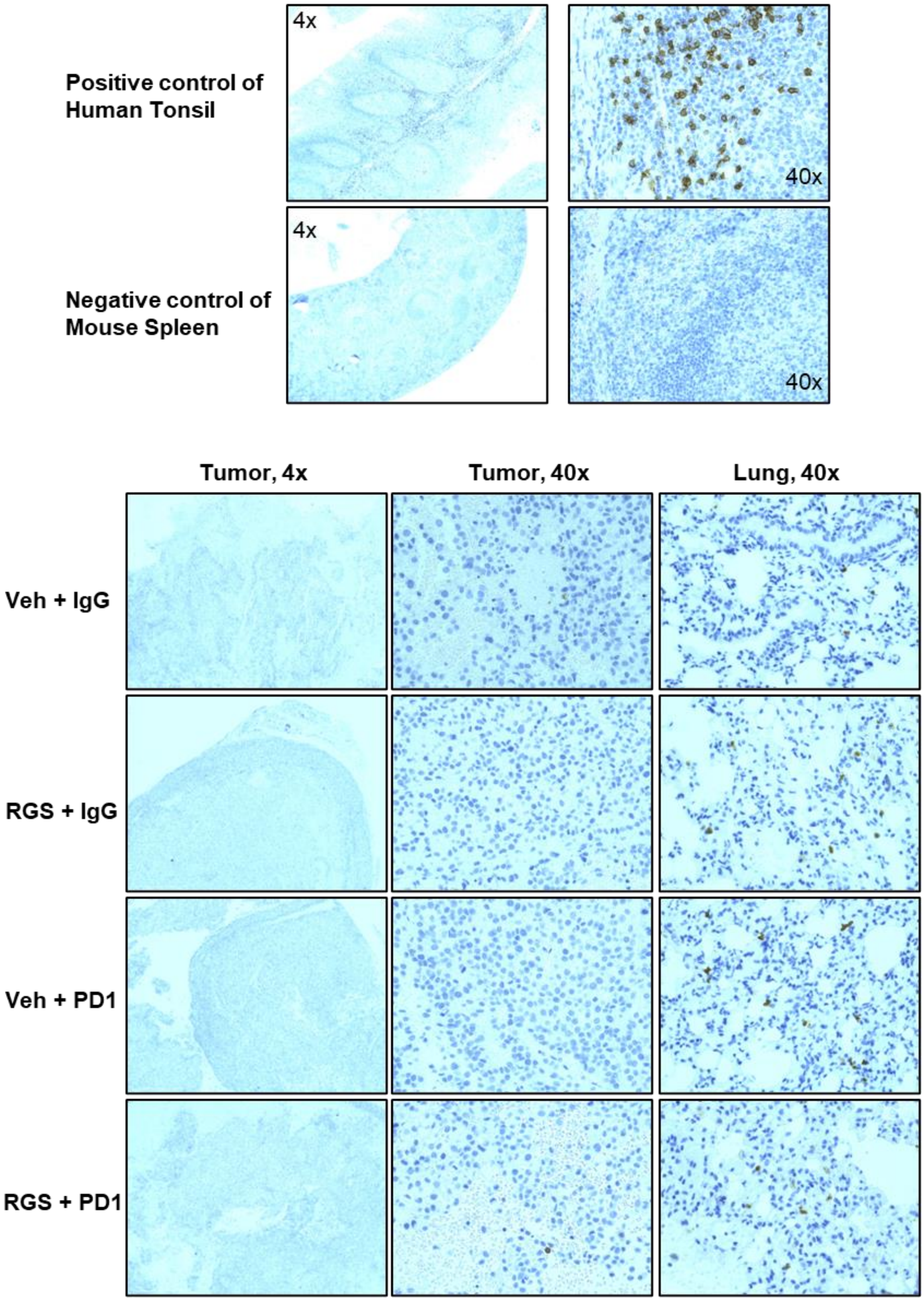

Supplementary Figure S5. Limited CD8 cell content in the tumor of huNOG-EXL-PDX mice bearing PDX-3101. Immunohistochemistry staining of human CD8+ cells in the indicated tissues. Results from human tonsil and mouse spleen were included as positive and negative controls, respectively.

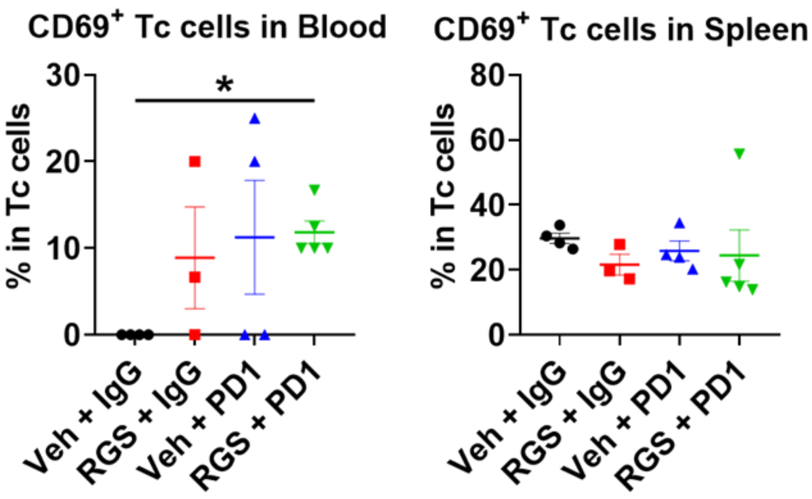

Supplementary Figure S6. Expression of activation marker CD69 on T cells from blood and spleens of huNOG-EXL-PDX mice bearing PDX-3101 tumors. Percentage of CD69+ cells based on flow cytometry data in the blood and spleen of individual humanized mice. Cells were gated on human CD45+CD3+CD8+ cytotoxic T (Tc) cells.

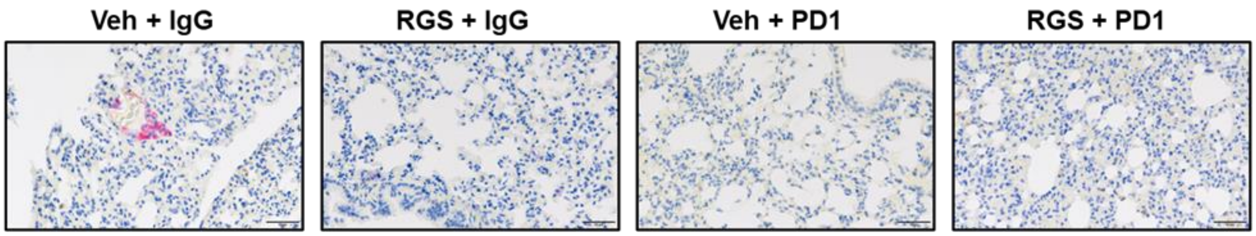

Supplementary Figure S7. Histologic evaluation of the lungs of huNOG-EXL mice bearing PDX-3101 tumors. The lung tissues of humanized mice demonstrated normal-appearing lung parenchyma. Immunohistochemical staining using a combined anti-human Melan A/Mart1 red stain for human melanoma cells demonstrated a small, 1mm, deposit of metastatic melanoma in the lung of a vehicle-treated mouse.

Supplementary Table S1. Antibodies for flow cytometry detection of human immune cells in humanized mice and human blood and mouse immune cells from wild type mice.

Target	Human Panel A	Clone	Fluorophor	Target	Human Panel C	Clone	Fluorophor
**All leukocytes, mouse**	mouse CD45	30-F11	Apc/Cy7	All leukocytes, mouse	mouse CD45	30-F11	FITC
**All leukocytes, human**	human CD45	HI30	AlexaFluor488	All leukocytes, human	human CD45	2D1	APC
**T cells**	CD3	UCHT1	Percp/Cy5.5	T cells	CD3	UCHT1	Pac. Orange
**B cells**	CD19	HIB19	Pe/Cy7	B cells	CD19	HIB19	BV711
**NK cells**	CD56	HCD56	PE	NK cells	CD56	5.1H11	Sp. NIR685
**T helpers**	CD4	RPA-T4	BV421	T helpers	CD4	SK3	PE/Fire 640
**Cytotoxic T cells**	CD8	SK1	BV605	Cytotoxic T cells	CD8	SK1	BV510
**Activation**	HLA-DR	L243	bv650	Myeloid cells	CD11b	ICRF44	Percp/Cy5.5
**Activation**	CD38	HIT2	bv785	Dendritic cells	CD11c	3.9	BV650
**Activation**	CD69	FN50	APC	Monocytes	CD14	M5E2	BV570
**Viability**	Live/Dead Aqua			PMN-MDSCs	CD15	HI98	eFluor 450
**Target**	**Human Panel B**			Ag-presenting cells	HLA-DR	L243	APC-Fire 750
**All leukocytes, mouse**	mouse CD45	30-F11	APC/Cy7	Macrophages	CD206	15-2	PE-Cy5
**All leukocytes, human**	human CD45	HI30	AlexaFluor488	Regulatory T cells	CD36	eBioNL07 (NL07)	PerCP-eFluor 710
**Antigen (Ag)-presenting cells**	HLA-DR	L243	BV650	Viability	Zombie NIR		
**Myeloid cells**	CD11b	M1/70	BV750				
**Granulocytes**	CD66b	6/40c	Pac. Blue				
**Dendritic cells**	CD11c	Bu15	PE				
**Dendritic cells**	CD123	6H6	APC				
**Monocytes**	CD14	63D3	AlexaFluor700				
**Macrophages**	CD64	10.1	PE/Cy7				
**Macrophages**	CD163	GHI/61	BV605				
**Target**	**Mouse panel**						
**All leukocytes, mouse**	mouse CD45	104	BUV395				
**T cells**	TCRβ	145-2C11	BV510				
**Activated T cells**	CD69	H1-2F3	BV605				
**Myeloid cells**	CD11b	M1/70	BV750				
**NK cells**	NK1.1	PK136	PE-Cy7				
**B cells**	B220	RA3-6B2	PE-Cy5				
